# Novel Robotic Platforms for the Accurate Sampling and Monitoring of Water Columns

**DOI:** 10.3390/s16091378

**Published:** 2016-08-29

**Authors:** Roemi Fernández, Andrey Apalkov, Manuel Armada

**Affiliations:** Centre for Automation and Robotics (CAR) CSIC-UPM, Carretera de Campo Real, Km. 0,200, La Poveda, Arganda del Rey, Madrid 28500, Spain; apalkov.andrey@gmail.com (A.A.); manuel.armada@csic.es (M.A.)

**Keywords:** water column, sampling, monitoring, underwater robotic platforms, profiler, accurate vertical positioning

## Abstract

The hydrosphere contains large amounts of suspended particulate material, including living and non-living material that can be found in different compositions and concentrations, and that can be composed of particles of different sizes. The study of this particulate material along water columns plays a key role in understanding a great variety of biological, chemical, and physical processes. This paper presents the conceptual design of two patented robotic platforms that have been conceived for carrying out studies of water properties at desired depths with very high accuracy in the vertical positioning. One platform has been specially designed for operating near to a reservoir bottom, while the other is intended to be used near the surface. Several experimental tests have been conducted in order to validate the proposed approaches.

## 1. Introduction

The ability to acquire data and collect samples along a water column of several cubic meters is an important requirement in the study and monitoring of numerous physical, chemical, and biological processes that take place in seas, lakes, and reservoirs [1,2]. For instance, analysis of the formation, transport, dissolution, and burial of particulate material is fundamental to comprehending biogeochemical cycles. The distribution and transport of the biotic components of suspended particles such as larvae, plankton, microbes, and viruses are very important in understanding aquatic ecosystems [3]. Spatial and temporal patterns in the flux of sinking organic matter are central to the identification of elemental dynamics and food webs in the ocean [4,5]. Systematic investigation of silicon contents and silicon isotopic compositions of dissolved Si and suspended particulate matter in rivers is quite useful for determining the constraints of the geological and biological process of the drainage areas and the general processes that govern the global silicon cycle [6]. Thus, in the literature, it is possible to find numerous investigations related to the properties of the water columns, such as [7,8,9,10,11,12,13,14]. 

However, the state of the art related to the equipment utilized for the collection of samples in such studies is basically reduced to two types of devices. The first one consists of a monolithic system (usually a CTD surrounded by a circular rosette of Niskin bottles) that is submerged in the water and made to descend to various predefined depths manually or automatically from a ship or a platform [15,16,17,18]. This solution is labour-intensive and can be very expensive from an economic standpoint, especially if it is carried out from a support vessel. Moreover, it has the disadvantage that the depth is given by the distance covered from the point where the device is located until the surface of the ship or the platform, so the accuracy of the depth of descent may be decreased by the influence of some factor such as the movement of the ship or platform by the waves, the weather, and the tides. This error may be even of several metres when sampling has to be carried out near the bottom or near the water surface. An additional disadvantage of this solution is that for collecting samples of water at various locations, it is necessary to carry out displacements of a large ship among these locations, which leads to higher fuel costs. Consequently, the process of monitoring and sampling collection is much more expensive. 

AUVs are the second most commonly used type of devices for water column sampling. Although these vehicles have evolved significantly, they still exhibit many limitations [19,20,21,22,23,24,25,26]. One of them, and perhaps the most relevant for the proposed application, is the fact that, when the process of collecting samples of water begins, the device loses its neutral buoyancy status, since it experiences a weight increment without a variation in its dimensions. Consequently, the vehicle starts to sink and it becomes necessary to compensate for this effect with the help of special actuators and the corresponding control system. In addition, the use of propellers should be disregarded in this case, given that the turbulence induced by them would alter the water samples for small-scale studies. If the response of the control system is not fast enough, the underwater robot will begin to oscillate, which would produce errors in depth measurements. 

Besides these two types of devices, it is also noteworthy to mention the vertical profilers, which are extensively used for data series collection throughout a water column. Some models consist of a buoyancy-driven platform that makes repeated automatic round trips up and down a taut-wire with a constant velocity. Measurements are carried out at predefined depths. Aqualog, a Russian oceanographic mobile profiler [27], and the McLane Moored Profiler [28] can be included in this group. Other models utilize variable buoyancy for system propulsion. This is the case with BSOP and APEX^®^. BSOP is an instrument platform that stations itself on the sea floor and ascends and descends autonomously to gather water column profile data. It uses an oil-based buoyancy control system to ascend and descend at speeds up to 0.5 meters per second [29]. APEX^®^ is an autonomous, neutrally buoyant drifter that uses a variable buoyancy engine to surface on a programmed schedule. Numerous variants for specific application have been developed from APEX^®^, such as the prototypes presented in [30,31]. The main disadvantage of vertical profilers is that their application is limited to data series acquisition; they cannot be utilized for collection of samples of water.

With the aim of providing a simple solution to the described problem, this article introduces the design of two patented platforms [32,33] that have been proposed for the automation of both the monitoring and the collection of samples at precise depths of the water column. One platform has been specially designed for operating near to a reservoir bottom [33], while the other is intended to be used near the water surface [32].

## 2. Materials and Methods 

This section details the design and the basic operating principles of the two platforms proposed for the accurate sampling and monitoring of the water column.

### 2.1. Underwater Platform Conceived for Operating near a Reservoir Bottom

The first platform is characterized by having a body divided into two parts, a lower and an upper one, connected by a toothed timing belt used as a cable. This setting allows for placing the lower part of the body at the bottom of the reservoir, lake, or on the ocean floor, and using it as a fixed reference for the positioning of the upper part of the body, which will be responsible for carrying out the process of monitoring and collection of samples. Therefore, rather than considering either the water surface or the surface of a ship or vessel as reference, a pre-set level with respect to the bottom will be taken as the base, so the displacements of the upper part of the body on the vertical axis are quantified with respect to that level. In this way, errors in the estimation of the real depth caused by the influence of waves, the variable pressure, and the effect of tides are greatly reduced.

**Figure 1:**
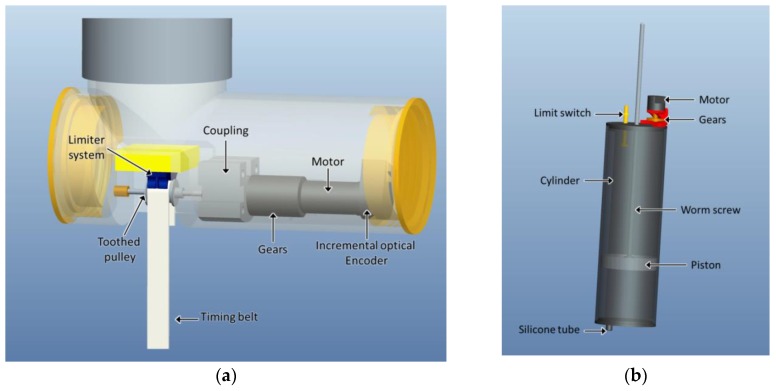
(**a**) Mechanism that allows the variation of the relative position of the upper part of the body with respect to the lower one; (**b**) ballast tank.

Since during the sampling process the lower part of the body will remain stationary at the bottom, and the upper part of the body will change its positioning, containers for water sampling are located in the upper part of the platform’s body so that collection and monitoring can be performed at programmed depths. Each of these containers is connected to the outside through a tube and a solenoid valve that is triggered by the control system. In addition to the batteries and the control system implemented in a PC/104, in this part of the body is also located the mechanism for the regulation of the effective length of the toothed timing belt used as a cable for connecting the lower and upper parts of the robotic platform. Therefore, this mechanism allows for variation of the relative position of the upper part of the body with respect to the lower one. To that end, the mechanism for regulating the effective length of the transmission element consists of an electric motor with gearbox, a toothed pulley installed on its axis, a limiter system, and an incremental optical encoder connected to the control system. Measurements acquired by the optical encoder are used by the control system to calculate the effective length of the transmission element. A basic outline of this mechanism is illustrated in Figure 1a. The limiter system of the toothed timing belt, which is implemented with a piece of metal that surrounds the pulley, is responsible for guiding the movement of the belt, hampering a possible jump of the same through the teeth of the pulley.

Therefore, once the baseline has been established by placing the lower part of the platform at the bottom, and knowing the effective length of the transmission element, it is possible to determine with high accuracy the depth where the upper part of the robot is located. A pressure sensor that provides depth information can also be used jointly with the proposed system in order to improve its robustness. 

The upper part of the body also includes a ballast tank. This ballast tank consists of a cylinder inside a piston driven by an electric motor and a transmission system. The cylinder communicates with the outside through a tube of silicone, so that when the motor activates the piston, some water is either introduced or ejected from the cylinder, depending on the direction of movement of the piston. This tank has the capacity to vary its volume to allow us to modify the buoyancy of the platform, so that it can ascend to the surface or descend to the bottom. The assembly further comprises two limit switches that make up a self-braking system for the piston and a potentiometer that allows for estimating the position of the piston inside the cylinder, and therefore the variation of volume achieved. Figure 1b shows the main elements that compose the ballast tank. Lastly, the lower part of the body has a sensor to detect contact with the bottom of the reservoir, lake, or ocean.

An aspect of paramount importance in the design process is the right choice of the relationship between the weights and volumes of the parts that make up the platform. These relations must ensure a secure contact of the lower part of the platform with the bottom and a certain tension of the timing belt for the sample collection. In addition, these relationships must allow the robot to come to the surface once the sample collection is completed. Therefore, the weights and volumes of the elements of the platform must satisfy the following conditions:

*V*_1_ + *V*_2_ = γ_1_/(*ρg*)·(*F*_1_ + *F*_2_ + *F*_3_ + *F*_4_) (1)

γ_2_*V*_3_ = *F*_3_/(*ρg*) (2)

*V*_1_ + *V*_2_ + *V*_3_ = γ_3_/(*ρg*)·(*F*_1_ + *F*_2_ + *F*_3_ + *F*_4_) (3)

γ_4_·(*V*_1_ + *V*_2_ + *V*_3_) = (*F*_1_ + *F*_2_ + *F*_3_ + *F*_5_)/(*ρg*) (4)

where *ρ* is the water density, *g* is the free fall acceleration, *V*_1_ is the external volume of the upper part of the body, *V*_2_ is the volume of the toothed timing belt, *V*_3_ is the external volume of the lower part of the body, *F*_1_ is the weight of the upper part of the body together with the empty containers for water samples and the empty ballast tank, *F*_2_ is the weight of the toothed timing belt, *F*_3_ is the weight of the lower part of the body, *F*_4_ is the total weight of the water that can be collected in the sampling containers, *F*_5_ is the weight of the water that can fill the ballast tank, and, finally, γ_1_, γ_2_, γ_3_, and γ_4_ are pre-set constants with a value greater than the unit that define a reserve of buoyancy (positive or negative) for the platform or its parts in certain conditions. Thus, the mathematical conditions are the following:

- The upper part of the platform should always have positive buoyancy.
- The lower part of the platform should always have negative buoyancy.
- The platform, as a whole, should have positive buoyancy if the ballast tank is empty.
- The platform, as a whole, should have negative buoyancy if the ballast tank is filled with water.
- Buoyancy, positive or negative, should always have a reserve margin.

The working principle of this platform is as follows. Initially, the containers for water samples are empty, the ballast tank is filled with air and the effective length of the toothed timing belt is the minimum possible. Under these conditions, when the platform is placed into the water, according to Equation (3), it exhibits positive buoyancy. Therefore, to start the operation, the piston is activated and the ballast tank is filled with water. Then, according to Equation (4), the platform starts its descent until the lower part of the body is positioned at the bottom. At this moment, the contact sensor transmits the corresponding signal to the control system. Next, the control system activates the mechanism that regulates the effective length of the belt by turning the toothed pulley with constant velocity. Thus, the condition given by Equation (1) is met, and the upper part of the body begins to emerge quickly enough to maintain a proper timing belt tension. The effective length of the belt continues to increase until the desired depth is reached for the first sampling. At this moment, the motor that rotates the pulley is disconnected and the solenoid valve of one of the containers for samples is opened. The valve remains open long enough for filling the container and then is closed. The last two steps are repeated for each depth in which either monitoring or collection of samples is required.

It is noteworthy that the process that regulates the length of the toothed timing belt can be halted if the loss of contact of the lower part of the body with the bottom is detected, and it can be restarted once the contact is recovered again. This ensures that the positioning of the upper part of the body with respect to the lower part of the body takes place only when the reference is fixed, which minimizes errors and increases the accuracy of the positioning of the upper part of the body at different depths.

Once all samples have been collected, the control system proceeds to move the piston of the ballast tank in such a way that the water contained therein is evacuated, reducing the weight of the platform. In this case, and in accordance with Equation (3), the Archimedes’ force that acts in the body of the platform would be greater than the weight of the platform. Thus, the platform would rise to the surface with the water samples collected. 

**Figure 2:**
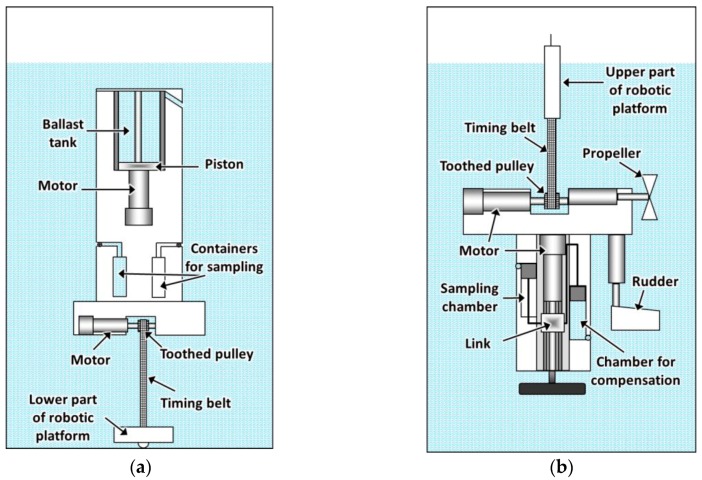
(**a**) Sketch of the robotic platform conceived for operating near a reservoir bottom; (**b**) sketch of the robotic platform conceived for operating near the water surface.

It is important to emphasize that there is a speed limitation in the change of the effective length of the belt. This limitation is related to the hydrodynamic friction forces that appear when parts of the platform move in the water. Thus, when the effective length of the toothed timing belt is increased with a speed much higher than the speed of ascent of the upper part of the body, the toothed timing belt can experience a deformation due to the lack of tension. In this case, the position estimated by the control system will not match the actual position of the upper part of the body, causing the sampling process to take place at imprecise depths. To prevent such errors, a sensor can be used to measure the tension of the toothed timing belt. Another option is to use a pressure sensor that detects the pressure variations instead of its exact value, that is, a sensor that detects the motion of the upper part of the body instead of the depth at which it is located. Thus, from the measurements acquired by this sensor it will be possible to determine if the upper part of the body is ascending (if the pressure measurements decrease continuously) or descending (if the pressure measurements increase continuously). It is also important to take into account that a considerable temperature variation can be experienced with the changes of depth. These temperature variations can produce expansion or contraction in the material that makes up the toothed timing belt, therefore modifying its length. Thus, to attain even more accuracy in the process of monitoring or sampling the column of water, the platform can have a sensor in the upper part of the body, which provides temperature measurements to the control system. These measurements acquired with the temperature sensor will come in handy to compensate for the variations in the length of the toothed timing belt, increasing the accuracy of the regulation mechanism. A sketch of the robotic platform is illustrated in Figure 2a. 

### 2.2. Platform Conceived for Operating Near the Water Surface

The second platform is also characterized by having a body divided into two parts, a lower and an upper part, connected by a toothed timing belt. However, this setting, unlike the previous one, uses the upper part of the body as a reference for the positioning of the lower part, which will be the responsible for carrying out the process of monitoring and collection of samples at programmed depths. For that, the containers for water sampling are located in the lower part of the platform’s body, as well as the mechanism for regulating the effective length of the toothed timing belt (see Figure 2b). Additionally, in this case, the lower part of the body will have negative buoyancy, the upper part will have positive buoyancy, and the whole platform will have a positive buoyancy of reduced value, in such a way that only a small fraction of the upper part of the body remains above the water surface. Therefore, the external volume of the lower part of the body is chosen so that it meets the condition *F*_1_ = *F*_2_ − *F*_3_, where *F*_1_ is the weight of the liquid in a volume equal to the external volume of the lower part of the body, *F*_2_ is the weight of the lower part of the body with the sampling containers empty, and *F*_3_ is a predefined value. In this way, the negative buoyancy of the lower part of the body is guaranteed if *F*_3_ > 0. The value of *F*_3_ will influence the speed of descent of the lower part of the body. The volume and weight of the upper part of the body are chosen in such a way that the condition *F*_4_ = *F*_5_ + *F*_6_ is satisfied, where *F*_4_ is the weight of the liquid in the volume equal to the external volume of the whole platform, *F*_5_ is the weight of the whole platform with the sampling containers full of liquid, and *F*_6_ is a predefined value. This ensures the positive buoyancy of the platform if *F*_6_ > 0. The value of *F*_6_ will determine the ascending speed of the lower part of the body.

The fact that a small fraction of the upper part of the body remains above the water surface allows for stabilizing the equilibrium position of the platform, but at the same time makes the upper part of the platform susceptible to external perturbations. These perturbations can be divided into random and deterministic. Influence of deterministic external factors such as tides can be compensated for by the control system, which should have information about tides depending on the time of the year. The influence of random effects, such as periodic waves on the surface, is compensated for by the special design of the upper part of the body, characterized by having a vertical element with a small horizontal cross-section. The mean area of this horizontal cross-section could be K times smaller than the mean area of the horizontal cross-section of the lower part of the body, where K is a predefined number and the volume of the vertical element is chosen such that *F*_4_ – *F*_7_ < *F*_5_, where *F*_7_ is the weight of the liquid in a volume equal to the volume of the vertical element. With this configuration, the upper part of the body will be close to the surface, but completely submerged, and only a part of the vertical element will be outside of the water. In this way, the upper part of the body dynamically behaves as an aperiodic link, capable of filtering the periodic external vertical forces. Thus, it is possible to significantly reduce the influence of sea waves in the upper part of the body, which in turn would affect the accurate vertical positioning of the lower part of the body.

**Figure 3:**
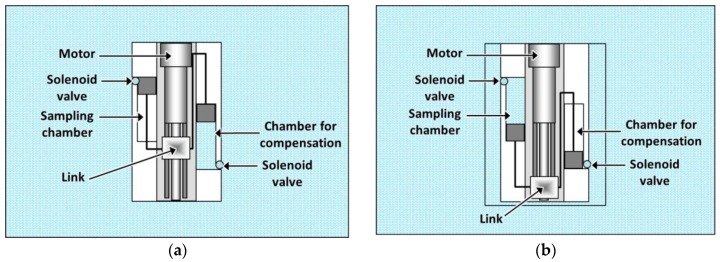
Sample container for the second prototype. (**a**) Before the water sampling process; (**b**) after the water sampling process.

On the other hand, this second platform should be capable of collecting samples of water without modifying its weight. On the contrary, the relationship between the Archimedes force and the weight of the platform would be decompensated, affecting the accuracy of the vertical positioning. For solving this problem, a special design has been conceived for the sampling containers (see Figure 3). In this way, each container consists of two parallel independent chambers, one empty and the other filled with liquid. In addition, each chamber is equipped with a piston, and these pistons are connected by a link that is activated by an electric motor. Thus, when the motor rotates, the pistons of both chambers move with the same velocity, and, consequently, the liquid entering the initially empty chamber is the same as that coming out from the initially filled chamber. With this design, the mass of the robotic platform, as well as its buoyancy, remain constant during the sampling process.

The upper part of the robotic platform could have a GPS connected to the control system in order to know the location of the platform in each moment. With the geographical coordinates provided by the GPS, some parameters of the control system that may vary considerably from one location on Earth to another, such as salinity, the acceleration of gravity, and tides, could be adjusted. The lower part of the body could also have a propeller and rudder connected to the control system so that the platform could autonomously carry out the sampling collection in different programmed zones. Even once the process of sampling is completed, it could return autonomously to a coast or a particular biological station. This concept can also be extended for the first prototype conceived for operating near a reservoir bottom.

Therefore, this second prototype could have two different operation modes: horizontal movement at the water’s surface to some predefined coordinates, and vertical movement of the lower part of the body for water sampling.

To start the process, the operator programs the coordinates and the depths at which the water samples will be collected in the control system of the platform. For the horizontal movement of the platform on the water surface, the lower part of the body is tightened to the upper part through a minimum effective length of the toothed timing belt. Then, the platform navigates to the desired location, guided by the GPS signal and with the help of the propeller and the rudder. Once the desired location is reached, it proceeds with the water sampling regime. 

In order to collect the sample of water at the desired depth, the effective length of the toothed timing belt is increased by the regulation mechanism until a predefined length *L*0 is attained. Since the lower part of the platform has negative buoyancy, it begins to sink. Then, the effective length of the toothed timing belt is reduced by the regulation mechanism until the moment the length corresponding to the desired depth is reached. After the control system has received the signal that the lower part of the platform is at the required depth, the collection of samples is carried out. For that, the DC motor of the specially designed water container displaces the link and consequently, moves the pistons that are located inside the chambers. This makes the piston in the chamber eject the water as compensation, whereas the piston in the empty chamber suctions the external water. In this way, the empty chamber is filled with the water sample. The steps above are repeated as many times as is necessary to perform the sampling of water at the desired depths. Once all samples have been collected, the effective length of the toothed timing belt is reduced to the minimum in order to bring them to the surface. Then, the robotic platform is ready to move to a new location in order to continue with the sampling process, or it can return autonomously to the base station.

## 3. Experimental Results and Discussion

**Figure 4:**
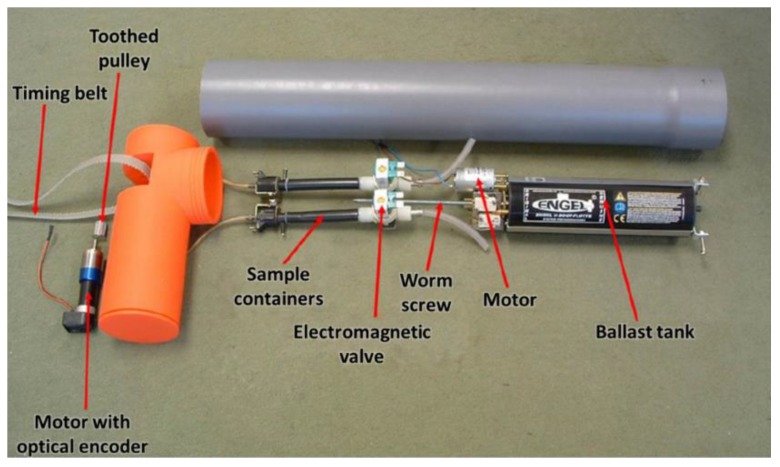
Upper part of the first prototype, manufactured for operating near a reservoir bottom.

In order to validate the conceptual design of the proposed platforms, two low-cost prototypes were manufactured and tested in an aquarium of 2 m height with a column of water of 1.8 m. The first prototype, which is conceived for operating near a reservoir bottom, has a mass of 6 kg and a height of 0.7 m. Figure 4 shows the main components of the upper part of this prototype.

**Figure 5:**
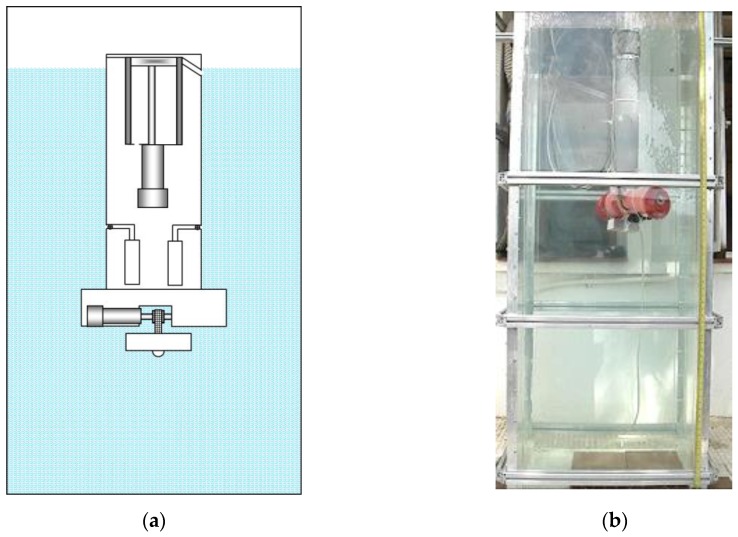
(**a**) Sketch of the first prototype in its initial position; (**b**) real prototype of the first robotic platform in its initial position.

**Figure 6:**
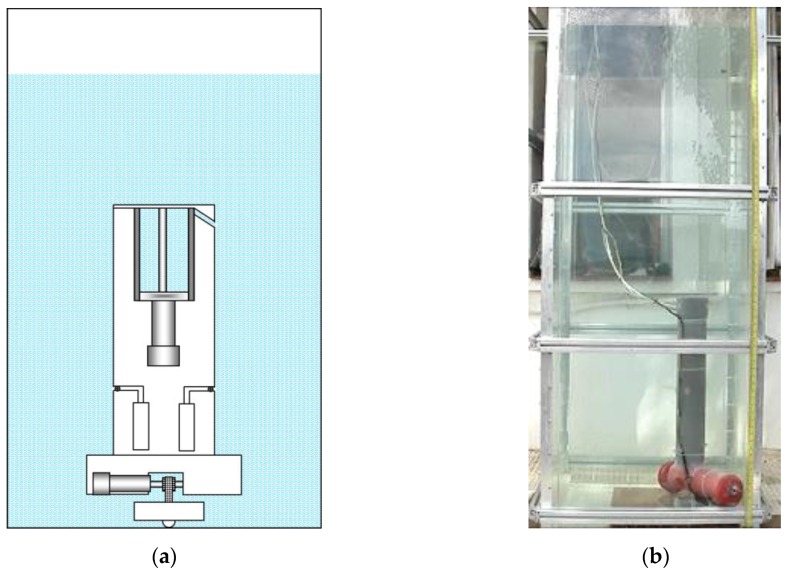
(**a**) Sketch of the first prototype at the bottom of the aquarium; (**b**) real prototype of the first robotic platform at the bottom of the aquarium.

**Figure 7:**
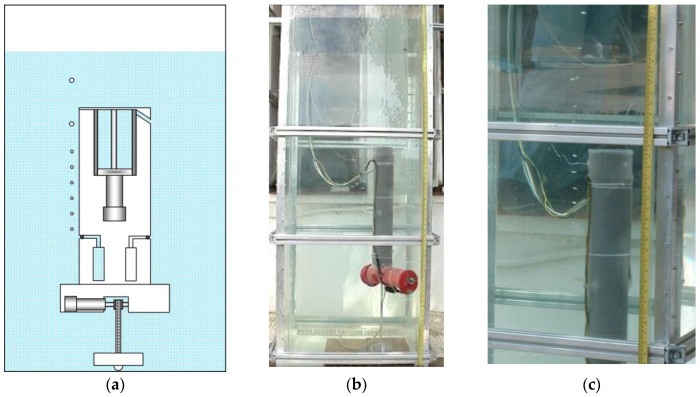
Moment when the first sample of water is collected. (**a**) Sketch of the prototype; (**b**) real prototype; (**c**) close-up view of the water sampling process.

**Figure 8:**
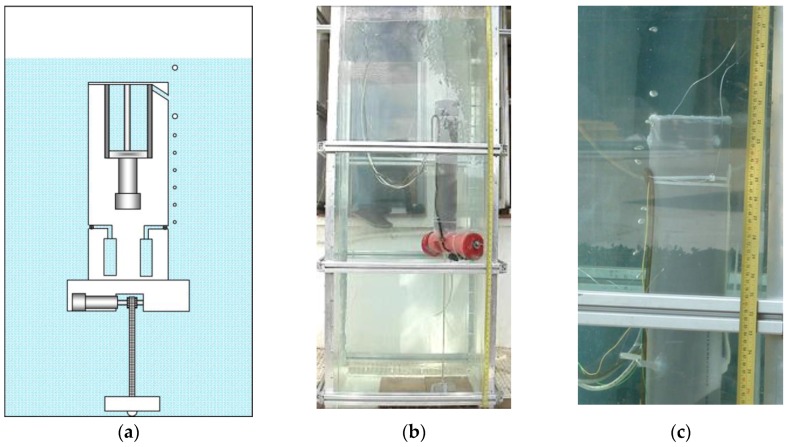
Moment when the second sample of water is collected. (**a**) Sketch of the prototype; (**b**) real prototype; (**c**) close-up view of the water sampling process.

**Figure 9:**
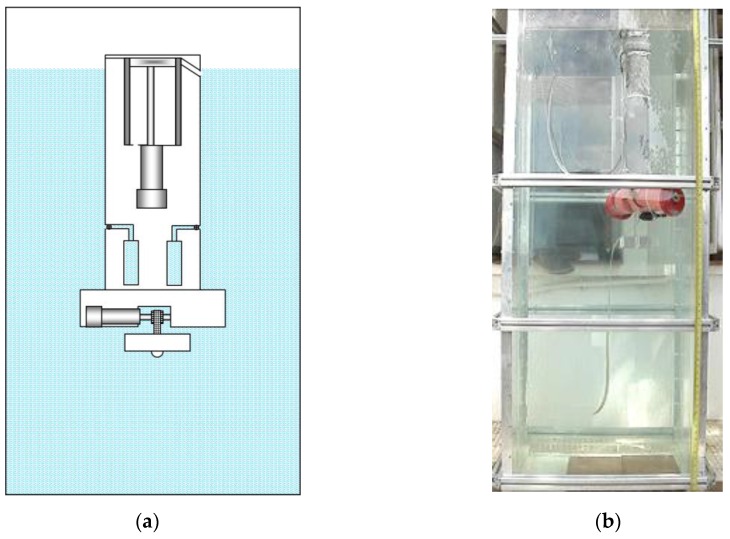
Status of the robotic platform once the working process has been completed. (**a**) Sketch of the prototype; (**b**) real prototype of the first robotic platform.

Figure 5, Figure 6, Figure 7, Figure 8 and Figure 9 display an experimental sequence that was carried out with the first manufactured prototype. Firstly, the containers for water sampling are empty, the piston of the ballast tank is in its highest position, and the toothed timing belt has the minimum effective length. According to Equation (3), the prototype exhibits positive buoyancy, as is shown in Figure 5.

Figure 6 shows the prototype when the lower part of its body is positioned at the bottom of the aquarium. This position is achieved after the piston is activated and the ballast tank is filled with water, which causes the descent of the platform, as described in Equation (4). 

Figure 7 shows the moment when the first sample of water is collected at the desired depth. For achieving this depth, the effective length of the toothed timing belt is regulated by the control system. As the condition given by Equation (1) is met, the upper part of the body emerges, tightening the toothed timing belt. When the desired depth is achieved, the electromagnetic valve of one of the containers is opened long enough to fill with the water sample, and then closed. It is possible to see in Figure 7 the air bubbles that come out of the container while it is filled with water. Figure 8 shows the collection of a second sample of water at a different depth. Finally, Figure 9 illustrates the last step, when the working process has been completed and the robotic platform rises to the surface with the water samples. This is possible thanks to the action of the control system that moves the piston in order to evacuate the water contained inside the ballast tank, which in turn causes the Archimedes’ force acting in the body to become greater than the weight of the robot, according to Equation (3). 

**Figure 10:**
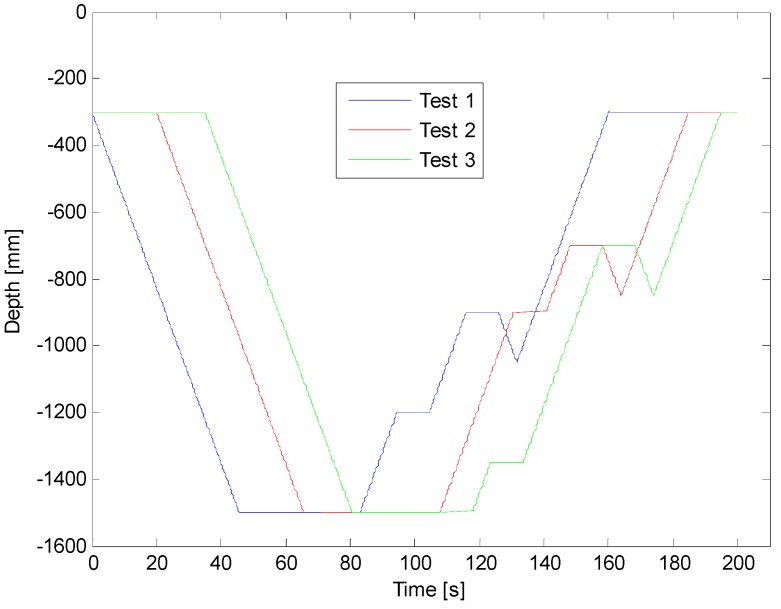
Trajectories followed by the first robotic platform in three different tests.

**Figure 11:**
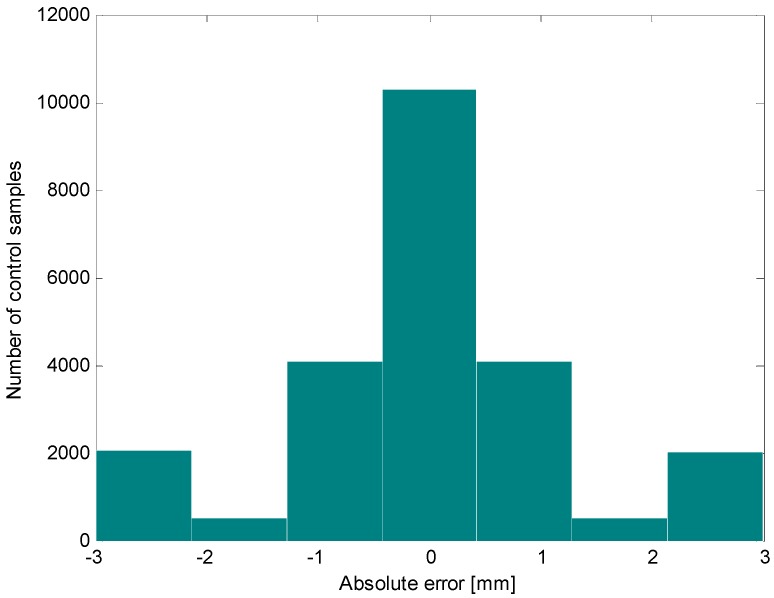
Distribution of positioning errors provided by the control system.

Figure 10 shows the trajectories followed by the first robotic platform in three different tests. In the first one, the platform was programmed to collect samples at 1200 and 900 mm, in the second test at 900 and 700 mm, and, lastly, in the third one, at 1350 and 700 mm. The depths displayed in Figure 10 indicate the vertical positioning of the sample containers achieved during the tests. The results of all experiments show that the robotic platform is quite accurate, with a maximum positioning error of ±3 mm (see Figure 11). This accuracy is limited only by the accuracy of the transmission between the motor and the toothed timing belt (errors in the position of teeth on belt, eccentricity of the pulley, and backlash between the toothed timing belt and the driving pinion). Other factors like waves, changes in atmospheric pressure, level of water in the aquarium, and changes in the mass of the robot, do not influence the accuracy of the upper part of the platform.

**Figure 12:**
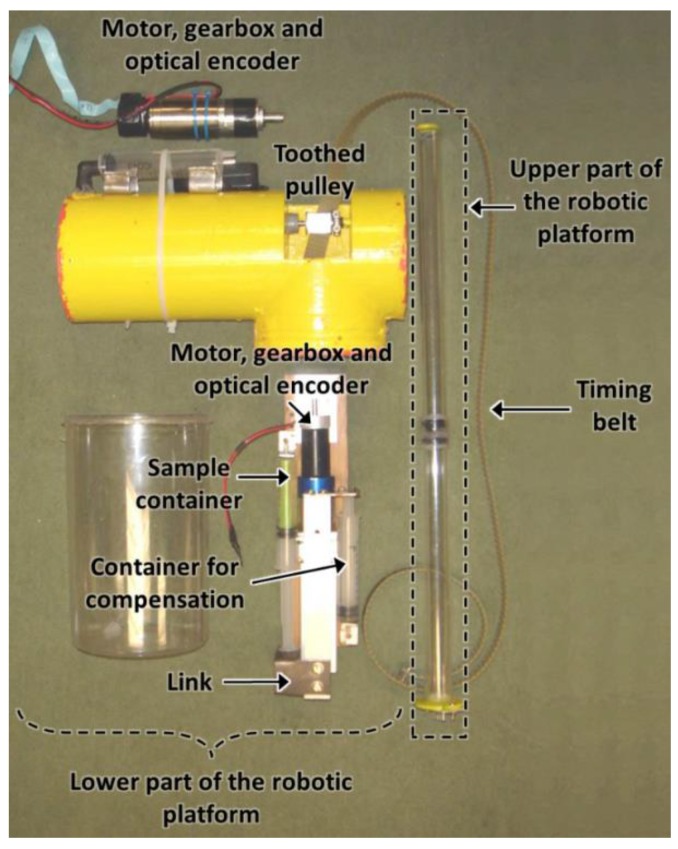
Main elements of the second manufactured prototype.

**Figure 13:**
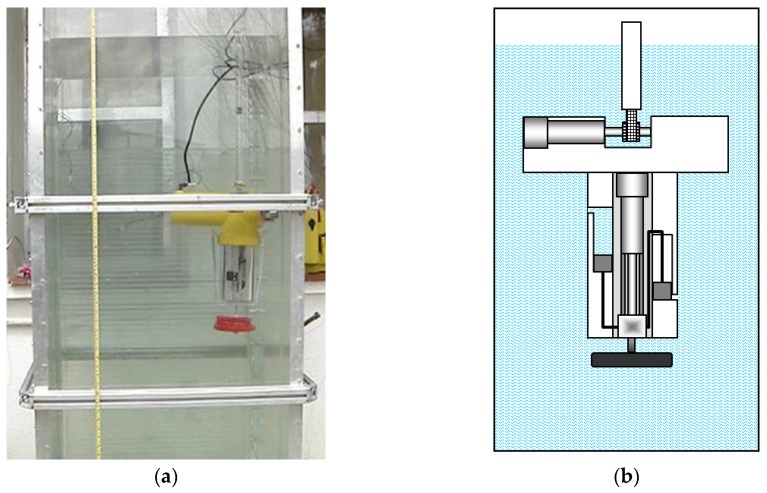
Initial state of the second robotic platform. (**a**) Real prototype of the second robotic platform; (**b**) sketch of the second prototype.

The main elements of the second manufactured prototype, which is designed for operating near the water surface, are shown in Figure 12. Figure 13 displays the initial state of the robotic platform. In this position, most of the robotic platform is underwater; just a small fraction of the upper part of the platform is above the water’s surface. In addition, the sampling chamber of the container is empty, the chamber for compensation is full of water, and the toothed timing belt has the minimum effective length.

**Figure 14:**
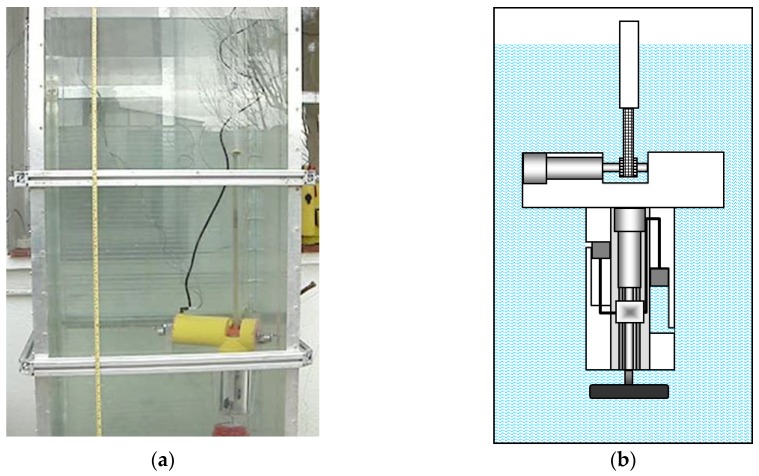
Moment when the first sample of water is collected with the second prototype. (**a**) Real prototype; (**b**) sketch of the second robotic platform.

Figure 14 shows the moment when the first sample of water is collected at the desired depth. For that, the effective length of the toothed timing belt is increased until the lower part of the body reaches the correct position. The upper part of the robotic platform does not modify its initial position. For the sampling process, the solenoid valves of both the sampling chamber and the chamber for compensation are simultaneously opened. Then the electromotor moves the link of the pistons in such a way that one of them pushes the water out of the chamber for compensation and the other takes the sample into the other chamber. In this way, the amount of water coming out of the compensation chamber is equal to the amount of water collected in the other chamber, and the total weight of the robot is not modified during the sampling. Therefore, the vertical positioning of the robotic platform is not affected during this process. 

Experimental tests demonstrated that this second prototype can achieve an accuracy of ±3 mm in the vertical positioning for sample collection, taking into consideration that the regulation of the effective length of the toothed timing belt is a quasi-static process, the robotic platform is in standing water, and the effective length of the toothed timing belt is less than one and a half meters. 

**Figure 15:**
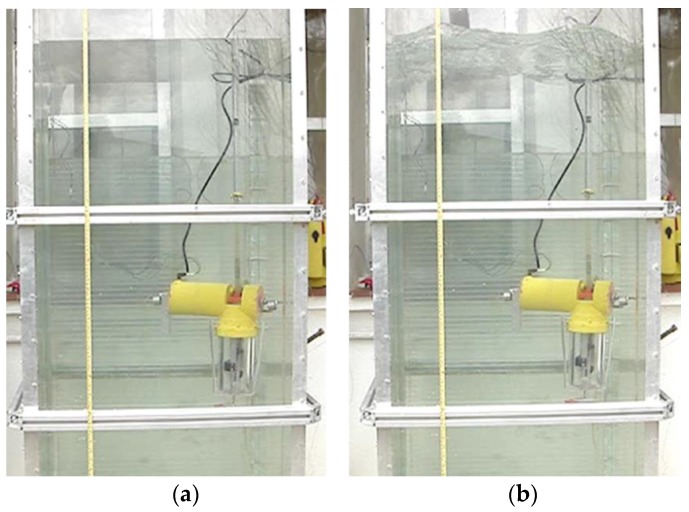
Test carried out to find out the influence of the surface waves on the accuracy of the vertical positioning of the robotic platform (**a**,**b**).

In addition, several experimental tests were carried out in order to find the influence of the waves on the water surface on the accuracy of the vertical positioning of the robotic platform. Results show that the smaller the cross-section of the upper part of the robotic platform, the lower the influence of the waves on the accuracy of the vertical positioning. As the top of our prototype has been made into a tube with a cross-section of 3 cm^2^, the oscillations that the robotic platform experienced vertically under the action of waves with amplitudes of up to 10 cm were only a few millimetres (see Figure 15). 

Despite the high accuracy achieved in the vertical positioning during the experimental tests conducted in lab conditions, it is important to mention that in real conditions (e.g., the robotic platforms operating in the sea), this accuracy may deteriorate due to the influence of factors such as:

- The presence of currents with different velocities along the water column, which can produce the inclination of the toothed timing belt, and consequently the inclination of the upper and/or the lower part of the robotic platform.
- The temperature dependence on the water depth of immersion, which can deform the toothed timing belt, modifying its length. These variations in water temperature can modify the effective length of the toothed timing belt in 1 cm.

In order to help the control system minimize such errors, the following mitigation measures are proposed:

- The installation of high-precision inclinometers in the lower and the upper part of the robotic platforms. Information provided by these sensors would enable the estimation of the tilt angle on the toothed timing belt and, consequently, would contribute to correct regulation of the effective length of the toothed timing belt for achieving the desired depth.
- The installation of a temperature sensor in the upper or the lower part of the robotic platform (in the upper part for the first robotic platform, and in the lower part for the second robotic platform). Taking into consideration the material the toothed timing belt is made of, information provided by this sensor can be used by the control system to compensate for the error introduced by the deformation of the belt.

Lastly, it is also important to mention that although most of the document is focused on describing the process of sample collection, the proposed prototypes can also be used for precise monitoring of the water column if they are endowed with the suitable sensors for that purpose.

## 4. Conclusions

The conceptual design of two robotic platforms devoted to the monitoring and collection of samples at accurate depths of the water column has been presented. One platform has been specially designed for operating near a reservoir bottom while the other is intended to be used near the surface. The presented solutions are simple and low-cost. In addition, the proposed conceptual designs can be adapted for a large number of applications that involve the study of water properties, taking into consideration the conditions given in Section 2.1 and Section 2.2.

In order to validate the feasibility of the proposed solutions, two simplified versions of the prototypes were manufactured and tested in an aquarium of 2 m height. Experimental results show that both robotic platforms are quite accurate in the vertical positioning for the collection of samples, exhibiting a maximum position error of 3 mm. Therefore, proposed designs are able to improve the accuracy of the current methods for water sample collection.

Future works will include the adaptation of the prototypes for their operation at greater depths, experimental tests with these new prototypes in real conditions, as well as the study of the influence of water currents and temperature variations in the accuracy of the vertical positioning. In addition, recent advances in the design and development of environmentally benign bio-fouling resistant coatings will be considered with the aim of finding a means to protect and prolong the lifetime of the toothed timing belt.

## Abbreviations

CTD: oceanography instrument used to determine the Conductivity, Temperature, and Depth of the ocean
AUV: Autonomous Underwater Vehicle
BSOP: Bottom Stationing Ocean Profiler
GPS: Global Positioning System

## References

[1] Gutiérrez M.H., Pantoja S., Quiñones R.A., González R.R. (2010). First record of filamnetous fungi in the coastal upwelling ecosystem off central Chile. Gayana.

[2] Silva A., Medeiros P.R., Silva M., Barbosa J. (2009). Diel vertical migration and distribution of zooplankton in a tropical Brazilian reservoir. Biotemas.

[3] Breier J.A., Rauch C.G., McCartney K., Toner B.M., Fakra S.C., White S.N., German C.R. (2009). A suspended-particle rosette multi-sampler for discrete biogeochemical sampling in low-particle-density waters. Deep Sea Res. I Oceanogr. Res. Pap..

[4] Dagg M.J., Jackson G.A., Checkley D.M. (2014). The distribution and vertical flux of fecal pellets from large zooplankton in monterey bay and coastal California. Deep Sea Res. I Oceanogr. Res. Pap..

[5] Wassmann P., Hansen L., Andreassen I.J., Riser C.W., Urban-Rich J., Båmstedt U. (1999). Distribution and sedimentation of faecal on the nordvestbanken shelf, northern Norway, in 1994. Sarsia.

[6] Ding T.P., Gao J.F., Tian S.H., Wang H.B., Li M. (2011). Silicon isotopic composition of dissolved silicon and suspended particulate matter in the yellow river, China, with implications for the global silicon cycle. Geochim. Cosmochim. Acta.

[7] Bezerra-Neto J.F., Pinto-Coelho R.M. (2007). Diel vertical migration of the copepod thermocyclops inversus (kiefer, 1936) in a tropical reservoir: The role of oxygen and the spatial overlap with chaoborus. Aquat. Ecol..

[8] Bianucci L., Fennel K., Denman K.L. (2012). Role of sediment denitrification in water column oxygen dynamics: ComParison of the north american east and west coasts. Biogeosciences.

[9] Dalsgaard T., De Brabandere L., Hall P.O.J. (2013). Denitrification in the water column of the central baltic sea. Geochim. Cosmochim. Acta.

[10] García-Moyano A., González-Toril E., Aguilera Á., Amils R. (2012). Comparative microbial ecology study of the sediments and the water column of the río tinto, an extreme acidic environment. FEMS Microbiol. Ecol..

[11] Gentz T., Damm E., Schneider von Deimling J., Mau S., McGinnis D.F., Schlüter M. (2014). A water column study of methane around gas flares located at the west spitsbergen continental margin. Cont. Shelf Res..

[12] González-DáVila M., Santana-Casiano J.M., Rueda M.J., Llinás O. (2010). The water column distribution of carbonate system variables at the estoc site from 1995 to 2004. Biogeosciences.

[13] Matijević S., Kušpilić G., Morović M., Grbec B., Bogner D., Skejić S., Veža J. (2009). Physical and chemical properties of the water column and sediments at sea bass/sea bream farm in the middle adriatic (Maslinova Bay). Acta Adriat..

[14] McDaniel M.D., David M.B., Royer T.V. (2009). Relationships between benthic sediments and water column phosphorus in illinois streams. J. Environ. Qual..

[15] Povinec P.P., Livingston H.D., Shima S., Aoyama M., Gastaud J., Goroncy I., Hirose K., Huynh-Ngoc L., Ikeuchi Y., Ito T. (2003). Iaea’97 expedition to the nw pacific ocean—Results of oceanographic and radionuclide investigations of the water column. Deep Sea Res. II Top. Stud. Oceanogr..

[16] Sauter E.J., Schlüter M., Wegner J., Labahn E. (2005). A routine device for high resolution bottom water sampling. J. Sea Res..

[17] Tesi T., Langone L., Goñi M.A., Turchetto M., Miserocchi S., Boldrin A. (2008). Source and composition of organic matter in the bari canyon (Italy): Dense water cascading versus particulate export from the upper ocean. Deep Sea Res. I Oceanogr. Res. Pap..

[18] Thomsen L., Graf G., Martens V., Steen E. (1994). An instrument for sampling water from the benthic boundary layer. Cont. Shelf Res..

[19] Asokan T., Seet G., Lau M., Low E. (2005). Optimum positioning of an underwater intervention robot to maximise workspace manipulability. Mechatronics.

[20] Evans M.E. (1993). A microstructure flux profiler control system design. IEEE Ocean. Eng. J..

[21] Grimble M.J., van der Molen G.M., Liceaga-Castro E. Submarine depth and pitch control. Proceedings of the Second IEEE Conference on Control Applications.

[22] Hayir A. (2003). The effects of variable speeds of a submarine block slide on near-field tsunami amplitudes. Ocean Eng..

[23] Kim T.W., Yuh J. (2004). Development of a real-time control architecture for a semi-autonomous underwater vehicle for intervention missions. Control Eng. Pract..

[24] McGookin E.W., Murray-Smith D.J. (2006). Submarine manoeuvring controllers’ optimisation using simulated annealing and genetic algorithms. Control Eng. Pract..

[25] Novick D.K., Pitzer R., Wilkers B., Crane C.D., de la Iglesia E., Doty K.L. The development of a highly maneuverable underwater vehicle. Proceedings of the Robotics 98: The 3rd International Conference and Exposition/Demonstration on Robotics for Challenging Environments.

[26] Wang S. (1986). Motions of a spherical submarine in waves. Ocean Eng..

[27] Ostrovskii A.G., Zatsepin A.G., Soloviev V.A., Tsibulsky A.L., Shvoev D.A. (2013). Autonomous system for vertical profiling of the marine environment at a moored station. Oceanology.

[28] Doherty K.W., Frye D.E., Liberatore S.P., Toole J.M. (1999). A moored profiling instrument*. J. Atmos. Ocean. Technol..

[29] Langebrake L.C., Lembke C.E., Weisberg R.H., Byrne R.H., Russell D.R., Tilbury G., Carr R. Design and initial results of a bottom stationing ocean profiler. Proceedings of the OCEANS ’02 MTS/IEEE.

[30] Sanford T.B., Dunlap J.H., Carlson J.A., Webb D.C., Girton J.B. Autonomous velocity and density profiler: EM-APEX. Proceedings of the IEEE/OES Eighth Working Conference on Current Measurement Technology.

[31] Johnson K.S., Coletti L.J., Jannasch H.W., Sakamoto C.M., Swift D.D., Riser S.C. (2013). Long-term nitrate measurements in the ocean using the in situ ultraviolet spectrophotometer: Sensor integration into the apex profiling float. J. Atmos. Ocean. Technol..

[32] Akinfiev T., Fernández R., Armada M. (2013). Dispositivo Para la Recolección de Muestras de Líquido y el Procedimiento Para su Control.

[33] Akinfiev T., Fernández R., Armada M. (2013). Aparato Para la Toma de Muestras de Líquido y el Procedimiento Para su Control.

